# Towards Eradication of PPR: Disease Status, Economic Cost and Perception of Veterinarians in Karnataka, India

**DOI:** 10.3390/ani13050778

**Published:** 2023-02-21

**Authors:** Gurrappa Naidu Govindaraj, Vinayagamurthy Balamurugan, Gundalahalli Bayyappa Manjunatha Reddy, Revanaiah Yogisharadhya, Timmareddy Sreenivasa Reddy, Gajalavarahalli Subbanna Naveenkumar, Kirubakaran Vinod Kumar, Hosahalli Rajanna Chaithra, Afrin Zainab Bi, Satya Parida, Felix Njeumi, Parimal Roy, Bibek Ranjan Shome

**Affiliations:** 1 ICAR, National Institute of Veterinary Epidemiology and Disease Informatics (NIVEDI), Ramagondanahalli, Yelahanka, Bengaluru 560064, India; 2 Animal Disease Surveillance Scheme, Department of Animal Husbandry and Veterinary Services, Government of Karnataka, Bengaluru 560024, India; 3 Food and Agriculture Organization of the United Nations (FAO), Viale delle Terme di Caracalla, 00153 Rome, Italy

**Keywords:** PPR incidence, eradication, financial viability of vaccination, Karnataka, India

## Abstract

**Simple Summary:**

Peste des petits ruminants (PPR) is a highly contagious animal disease affecting small ruminants that causes high morbidity and mortality. To prevent outbreaks, Karnataka state, India, has implemented the PPR-Control programme (PPR-CP) with a ‘mass vaccination’ strategy since 2010–11, resulting in a significant reduction in the number of outbreaks. However, the state continues to report outbreaks every year due to various reasons. Presently, the state is planning to eradicate the disease by 2025–26 by employing a new mass vaccination programme in coordination with the government of India’s PPR eradication plan. In this study, we report on the current status of PPR, its economic cost, the financial viability of vaccination plans, and the perspectives of field veterinarians in controlling and eventually eradicating the disease in Karnataka state. The disease incidence in the state declined significantly due to the implementation of mass vaccination and the benefits of vaccination outweighed the cost many-fold. The majority of the veterinarians concurred with the various activities of PPR-CP but a few indicated disagreement with the plan per se, the coordination between the functionaries, the available funding and the acceptance of the programme by farmers.

**Abstract:**

In this study, we assessed the PPR disease status, its economic cost, the financial viability of vaccination, and the perspectives of field veterinarians on the PPR vaccination programme implemented in Karnataka state, India. In addition to secondary data, cross-sectional surveys undertaken during 2016–17 (survey I) and 2018–19 (survey II) from 673 sheep and goat flocks and data collected from 62 veterinarians were analysed. The economic costs and perceptions of veterinarians were analysed using deterministic models and the Likert scale, respectively, and the financial viability of vaccination programmes under the best (15%), base (20%), and worst-case (25%) PPR incidence scenarios, considering two different vaccination plans (plan I and plan II), was assessed. The disease incidence in sheep and goats was found to be 9.8% and 4.8% in survey I and survey II, respectively. In consonance with the increased vaccination coverage, the number of reported PPR outbreaks in the state declined significantly. The estimated farm-level loss of PPR varied between the surveyed years. Even under the best-incidence scenario, under vaccination plan-I and plan-II, the estimated benefit–cost ratio (18.4:1; 19.7:1), the net present value (USD 932 million; USD 936 million) and the internal rate of return (412%) implied that the vaccination programmes were financially viable and the benefits outweighed the cost. Though the majority of veterinarians perceived that the control programme was well planned and rolled out in the state, a few of them disagreed or were neutral towards the plan per se, towards the coordination between functionaries, the availability of funding, and the programme acceptance by farmers. Despite many years of vaccination, PPR still persists in the Karnataka state for various reasons and in order to eradicate the disease, a review of the existing control programme with strong facilitation from the federal government is needed.

## 1. Introduction

Peste des Petits Ruminants (PPR) is an acute febrile viral disease of sheep and goats and is generally referred to as ‘sheep and goat plague’. The disease is characterised by mucopurulent nasal and ocular discharges, necrotising and erosive stomatitis, enteritis, diarrhoea, and bronchopneumonia [1,2]. The causative agent, PPR virus (PPRV), belongs to the genus *Morbillivirus* under the family Paramyxoviridae. It is closely related to the Rinderpest virus that causes cattle plague, which was eradicated globally by 2011. The disease was first reported in sheep and goats in Côte d’Ivoire, West Africa in 1942 [3] and later spread to different countries in Asia and Africa. The global annual loss due to PPR is estimated to range from USD 1.45 billion to USD 2.1 billion [4]. Small ruminants are an important asset of landless, marginal and small farmers and disease outbreaks in flocks undermines the food and livelihood security of these poor farmers. Hence, considering the importance of PPR for small holder’s, the Food and Agriculture Organisation (FAO) and the World Organisation of Animal Health (WOAH) launched a global plan to eradicate PPR by 2030 [4].

In India, the disease was first reported in 1987 in a small sheep flock in the village of Arasur in Tamil Nadu state, which was caused by a lineage III virus [5]. Subsequently, large outbreaks were reported in 1994–1995, which was caused by a lineage IV virus. Later, the disease spread to the entire country, and now PPR is enzootic in India and outbreaks are reported regularly among small ruminant population [1,2,6,7,8]. Very high rates of morbidity and mortality (> 50 percent) due to high fever, pneumonia, diarrhoea, and dehydration have been reported in the affected flocks [9]. Epidemics have enormous consequences on livestock productivity and affect the livelihoods of farmers and associated stakeholders in the sheep and goat value chain. Outbreaks have been reported regularly in the majority of the states of India since 2002. The losses due to PPR, as estimated by various researchers in India during different time periods, were INR 16116 million (USD 230.2 million) during 2015 [10], INR 88,951 million (USD 1270.7 million) based on reported outbreaks during 2008 to 2012 [11], and INR 45,710 to 46,830 million (USD 653–669 million) during 2016 [12]. Considering the devastating nature of the disease, an effective Vero-cell-line-based live attenuated indigenous freeze-dried vaccine (Sungri 96) with a shelf life of more than one year at 4 °C, which provides immunity for three to six years, was developed by the ICAR–Indian Veterinary Research Institute [13,14]. In India, since 2002, focused vaccinations of sheep and goats have been undertaken, and since 2010–2011, in southern states, the PPR-Control Programme (PPR-CP), with mass vaccination drives, has been implemented. This programme was implemented even before the global framework to control and eradicate PPR was developed. The Department of Animal Husbandry, Dairying and Fisheries (DADF), Government of India, sponsors the PPR-Control Programme (PPR-CP) in India and the participating states adopted a plan to carry out 100% vaccination of the risk population of small ruminants in the first year, followed by 30% bi-annual vaccination in the subsequent years to cover the naïve population [2,15]. Among the southern states, Karnataka and its contiguous state, undivided Andhra Pradesh, have implemented focused vaccination since 2002 and carried out mass vaccinations during 2004 and 2007–2008, respectively. Thereafter, they conducted annual vaccination programmes until 2010. After the first phase of the implementation of PPR-CP in these states during 2010–11, a significant reduction in the number of outbreaks in Karnataka state and a 99% reduction in PPR burden, with a flock immunity level of 81% to 95.6%, was observed in undivided Andhra Pradesh [2]. This was achieved due to the adoption of a systematic vaccination programme under PPR-CP. In central India, Chhattisgarh state has carried out annual mass vaccination campaigns (MVCs) since 2010 on the ‘pulse polio vaccination mode’ for a designated period of 11–12 days in a year, resulting in no PPR outbreaks having been reported in the state since 2013–14 [2,16]. In the second phase of PPR-CP, since 2015, although the rest of India’s states and Union Territories (UTs) have implemented PPR-CP [2], the continuation of the programme is largely elusive in these states.

Karnataka accounts for 17.21 million sheep and goats.PPR is endemic in the state and is the single largest cause of mortality in small ruminants. Hence, to control the disease, ‘focused vaccination’ has been undertaken since 2003–2004, and the PPR-CP strategy, including ‘mass vaccination’, was implemented in 2010–2011 [10,15]. The main objectives of PPR-CP are 100% vaccination coverage, with the aim of bringing the outbreaks and epizootics to the level of zero and to make the state a PPR-free zone. The number of outbreaks has significantly decrease in the state due to the implementation of the vaccination programme. Now the state has planned to eradicate the disease by 2025–2026, implementing a further planned mass vaccination programme in consonance with the government of India’s recent PPR eradication plan. In general, the economic costs and financial effectiveness of PPR vaccination programmes are limited in PPR-endemic countries, including India. Hence, the present study was undertaken to understand the disease status after implementing the vaccination programme, vaccination coverage vis-à-vis outbreaks, the economic cost of the disease, the financial viability of the vaccination programme, and the perceptions of veterinarians in the PPR-endemic state of Karnataka, India.

## 2. Materials and Methods

### 2.1. Study Area

**Figure 1:**
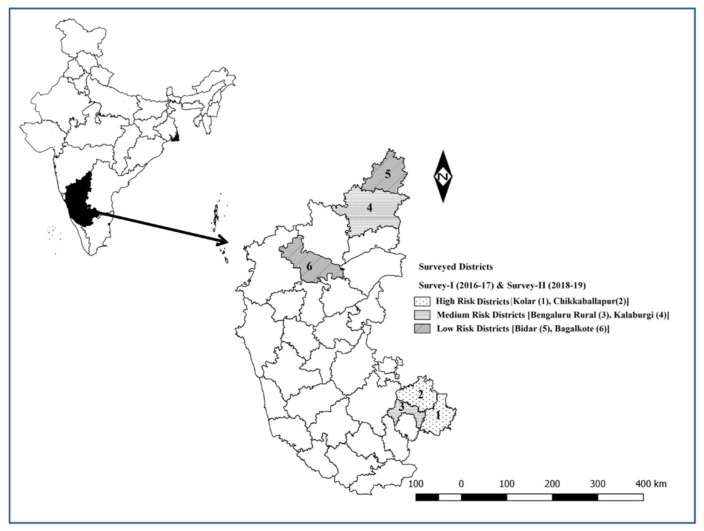
Districts with a high risk (Kolar (1), Chikkaballapur (2)), medium risk (Bangalore rural (3), Kalaburgi (4)), and low risk (Bidar (5), Bagalkote (6)) surveyed in Karnataka during survey I (2016–2017) and survey II (2018–2019).

Karnataka is the 6th largest state, comprising 30 districts, which cover an area of 191,791 square kilometers or 5.83% of the total geographical area of India. Karnataka is the only southern state to have land borders with all of the other six southern Indian sister states. It is bordered by the Arabian Sea to the west, Goa to the northwest, Maharashtra to the north, Telangana to the northeast, Andhra Pradesh to the east, Tamil Nadu to the southeast, and Kerala to the south. As per the 20th livestock census, 2019, the total livestock population in Karnataka state was 29 million, of which 59.35% (17.21 million) comprised sheep and goat, of which, sheep constitute 64.21% (11.05 million). To understand the PPR incidence and the disease cost, the primary survey was undertaken during 2016–2017 and 2018–2019 and the geographical locations of the survey districts are presented in Figure 1.

### 2.2. Sampling Procedure

Cross-sectional surveys were undertaken in six districts of Karnataka state among sheep- and goat-rearing households during 2016–2017 (survey I) and 2018–2019 (survey II). Initially, the districts in the state were grouped into three risk groups (high, medium, and low) based on the cumulative number of outbreaks that occurred in the districts in the three years preceding 2016–2017, the total number of PPR attacks per 1000 heads of sheep and goats, and the frequency of outbreaks and livestock density per 100 km^2^, and one district was selected in each of the risk group for the primary survey. During survey I, three districts, Kolar (high PPR risk), Bangalore rural (medium PPR risk), and Bagalkote (Low PPR risk), and in survey II, three districts, Chikkaballapura (high PPR risk), Kalaburgi (medium PPR risk) and Bidar (low PPR risk), were surveyed. A multistage random sampling procedure was followed to conduct survey I and survey II. In the first stage, one district from each of the risk groups was selected randomly and in the second stage 2–4 blocks (a block is a smaller administrative unit in a district and each district may comprise 2 to 5 or more blocks, depending on the population of the district) were randomly selected in each of the selected districts. In the third stage, in each selected block, one veterinary dispensary/hospital was selected randomly. In the fourth stage, the sheep- and goat-rearing villages under the jurisdiction of the dispensary/hospital were enlisted, of which 5–10 villages were selected randomly. In the final stage, in each of the identified villages, the individual farmers/households were selected randomly for the primary survey. The number of flocks to be surveyed in the identified villages was determined based on the proportion of sheep- and goat-rearing households in the village.

### 2.3. Sample Size

The sample size for the primary survey was estimated as below [17],

(1) $$\mathrm{SS}=\frac{z^{2}\left(p\right)\left(1-p\right)}{e^{2}}$$

where SS is the required sample size; Z is the z-value at a 95% confidence interval (1.96); p is the proportion of sheep- and goat-rearing households to the total number of households rearing livestock (0.224), and e is the acceptable sampling error (5%). The estimated total sample size for the survey was 267 sheep- and goat-rearing flocks. However, the primary data was collected from 350 flocks during 2016–2017 (survey I) and 323 flocks during 2018–2019 (survey II) using pre-tested schedules.

### 2.4. Data Collection and Identification of PPR-Affected Flocks

The primary data on socio-economic parameters, sheep and goat inventories, production parameters, morbidity and mortality, PPR vaccination and treatment cost, etc. were collected from the selected sheep and goat farmers in the surveyed villages as per the sampling plan. Among the sampled farmers, photographs of various clinical signs of PPR [18] were shown to the farmers and based on their observations of clinical signs, the flocks were diagnosed and grouped into PPR-affected and non-affected groups. Furthermore, the PPR incidence reported by the farmers during the survey was triangulated with the data provided by jurisdictional veterinary doctors to confirm the occurrence of PPR in the surveyed flocks, as these veterinarians are the ‘gatekeepers’ for the animal diseases occurring in their jurisdiction.

### 2.5. Estimation of the Flock-Level Economic Cost of PPR

Sheep and goat inventory, clinically diagnosed PPR cases, and deaths were considered in order to estimate the primary metrics of morbidity and mortality levels. The mortality loss was estimated based on the number of animals that died in each age and sex group multiplied by the market value of the apparently healthy animals and subtracting the recovered value of the animal, if any. To calculate the weight loss after PPR outbreaks, measurements of the pre- and post- disease weights of the animals across the breed, age, and sex were required. As this was a cross-sectional survey-based study, on the day of the survey, it was possible that diseased flocks may not have been encountered. Hence, to calculate the weight loss due to PPR, the actual weight of the animals of various breeds, ages, and sexes from the apparently healthy flocks were documented by physically weighing the animals during the survey. A conservative 10% reduction (although a 15% weight reduction for sheep and goat pox was reported earlier [19]) in the recorded weights for the healthy flocks was considered as the weight loss in the PPR-affected flocks, and accordingly, the weight loss was calculated across breed, age, and sex for the flocks reported to be affected by PPR. The distress sale loss was calculated based on the number of animals sold under distress and the difference in the actual price obtained during a healthy state and the distress sale value of the animals. The treatment cost was calculated based on the expenditure made for drugs/medicines and veterinarian charges during the PPR outbreak in the flock and converted to the per-animal cost. Similarly, the opportunity cost of labour engaged for nursing the sick animals was calculated based on the incremental labour hours spent by the farm family or hired members multiplied by the prevailing wage rate in the PPR-affected villages. After calculating the loss per flock, appropriate weights were considered based on the number of animals that died and that were infected and recovered among the sheep and goats reared by the sample farmers to calculate the per-animal loss. The market value/prices of animals of various age and sex groups and labour wage rate prevailing in the surveyed villages during the survey periods (2016–2017 and 2018–2019) were considered in order to estimate the various losses. The estimated disease cost per animal during survey I (2016–2017) was converted to the 2018–2019 constant price, based on the prevailing consumer price index during the respective years [20].

### 2.6. Estimation of the Financial Viability of Vaccination

#### 2.6.1. Vaccination Period

The cash-flows were estimated in order to assess the benefit versus the cost of the vaccination programme from the initial year of PPR vaccination in Karnataka (2003–2004) up to the year selected for the potential control and eradication of PPR (2025–2026) as envisaged in the draft plan for PPR eradication in India. The vaccination period was divided into two stages—vaccination before PPR-CP implementation in the state (from 2005–2006 to 2010–2011) and vaccination after PPR-CP implementation (2011–2012 to 2025–2026).

#### 2.6.2. Vaccination Coverage and Plans

The data on the number of sheep and goats vaccinated in Karnataka state were collected from secondary sources from 2003–2004 to 2020–2021 in order to calculate the vaccine coverage (%). The benefits and costs of vaccination were estimated under two methods of vaccination plans. The first plan involved actual vaccination coverage from 2003–2004 to 2020–2021, covering 100% of the risk population from 2021–2022 to 2023–2024, followed by the need-based vaccination of 10% for the next two years (2024–2025 and 2025–2026) and the second plan was as per the PPR-CP plan, i.e., the actual vaccination coverage from 2003–2004 to 2020–2021 and the vaccination of 100% of the at-risk population during 2021–2022, followed by 30% bi-annual coverage for the two subsequent years (2022–2023 to 2023–2024) and 10% need-based vaccination coverage for next two years (2024–2025 and 2025–2026).

#### 2.6.3. Population Projection

The livestock population census in Karnataka was available only for quinquennial periods (i.e., 2003, 2007, 2012, and 2019) and hence to calculate the sheep and goat population in Karnataka for in-between years, the interpolation method was employed, using the compound annual growth rate (CAGR) of the sheep and goat population between respective livestock censuses. Furthermore, to predict the population for the period from 2020–2021 to 2025–2026, the average CAGR of the sheep and goat population between the 2003 and 2019 livestock censuses was considered.

#### 2.6.4. Incidence Interpolation

The PPR incidence levels with and without vaccination intervention were crucial for our financial evaluations. Due to various surveillance bottlenecks in developing countries, the reported disease incidence differs from the field-level incidence. For Karnataka state, the PPR incidence in the field based on surveys was available only for two years (2016–2017; 2018–2019). Hence, the 8% incidence level reported in the literature [21] during 2008–2009 for Madhya Pradesh state was considered as the initial incidence level in the study state (Karnataka) for the first year of financial assessment (i.e., 2003–2004). Furthermore, in determining the PPR incidence under a scenario with a vaccination intervention, we assumed that this 8% incidence level increased gradually and reached 12% during 2010–2011, as the progress of vaccination in the initial years was very slow due to the adoption of the ‘focussed vaccination’ policy in the state. Thereafter, mainly after PPR-CP implementation (since 2010–2011), the incidence level gradually decreased due to the adoption of the ‘mass vaccination’ drive. In the primary surveys undertaken in this study, we estimated an incidence of 9.8% and 4.8% in Karnataka during 2016–2017 and 2018–2019, respectively. Considering the initial incidence (2003–2004), the incidence under focussed vaccination (2010–2011), and the estimated incidence after PPR-CP implementation (2016–2017 and 2018–2019), the possible incidence levels in in-between years under the vaccination scenario were derived via interpolation through the linear method. At the end of 2023–24, as per the draft national strategic plan, 100% of the sheep and goat population [2] will be vaccinated, and as a result, the disease incidence will be very low during 2023–2025 to 2025–2026. Hence, for the financial analysis, a PPR incidence of zero was assumed during 2023–2025 to 2025–2026 (In India, the financial year lasts from April until March of the following year (for example, the year 2010–2011 year represents the period from April 2010 to March 2011). Similarly, the stream of incidences in different years between 2003–2004 and 2025–2026 under the scenario without vaccination intervention was derived via interpolation through the linear method. The three possible incidence scenarios (Supplementary Table S1) were as follows: an 8% incidence level during 2003–2004 that increased to 15% by 2010–2011 and remained at the same level until 2025–2026 (low incidence); an 8% incidence level during 2003–2004 that increased to 20% by 2025–2026 (medium incidence); and an 8% incidence level during 2003–2004 which increased to 25% by 2025–2026 (high incidence). These differences between incidence levels under scenarios with and without vaccination in different years were considered in order to estimate the avoided costs/benefits of morbidity and mortality reductions that occurred due to PPR vaccination. The details of interpolated incidence levels in scenarios with and without vaccination (with three possible incidences under without vaccination) are presented in Supplementary Table S1.

#### 2.6.5. Benefit Stream

The benefits of vaccination in different years were calculated based on the difference in the PPR incidence (the disease avoidance level) under scenarios with and without vaccination, the projected risk population in the respective year, the per-animal disease cost, the morbidity and mortality levels, and the effectiveness of vaccination (80%). The disease cost included mortality, body weight reductions, distress sales, treatment costs, and the opportunity cost of labour per animal due to PPR. The disease cost, incidence, morbidity, mortality, and distress sale proportions were calculated based on the data collected in the primary surveys undertaken in Karnataka state during 2016–17 and 2018–19. An annual 3% deflation rate (from 2003–2004 to 2017–2018) and inflation rate (from 2019–2020 to 2025–2026) were applied [22] to calculate disease cost per animal at current prices.

The total benefit of vaccination against PPR was calculated as follows.

(2) $$B_{v}=\sum_{i=1}^{n}\left\{\left[(\Delta I_{i}{\times P}_{i})\;\left\{\left({\mathrm{Mp}}_{i}{\times \mathrm{Lm}}_{i}\right)+\left({\mathrm{Wp}}_{i}{\times \mathrm{Lw}}_{i}\right)+\left({\mathrm{Dp}}_{i}{\times \mathrm{Dl}}_{i}\right){+\mathrm{Tc}}_{i}{+\mathrm{Oc}}_{i}\right\}\right]{\times V}_{\mathrm{Ei}}\right\}$$

where Bv = total benefits of vaccination/PPR avoidance cost (USD); (ΔI)i = difference in PPR incidence under scenarios with and without vaccination in the ith year (%), Pi = projected population in the ith year (No.), Mpi = proportion of mortality due to PPR in the ith year (%), Lmi = average mortality loss per animal in the ith year (USD), Wpi = proportion of morbidity due to PPR in the ith year (%), Lwi = average body weight reduction loss per animal in a PPR-recovered animal in the ith year (USD), Dpi = distress sale proportion due to PPR in the ith year (%), Dli = average distress sale loss per animal in the ith year (USD), Tci = average treatment cost per animal in the ith year (USD), Oci = average opportunity cost of labour per animal in the ith year (USD), VEi = vaccination effectiveness in the ith year (80%), and n = number of years (i = 1, 2, 3, …n).

#### 2.6.6. Vaccination Cost Stream

The total vaccination cost per annum was calculated based on the proportion of sheep and goats vaccinated, multiplied by the vaccination cost (vaccine cost per dose and the vaccination logistics and accessories cost per dose/animal). The vaccine cost and vaccination logistics and accessories cost varied depending on the vaccination strategy adopted, viz., focussed/routine vaccination or using the PPR-CP model. In Karnataka, the focussed/routine vaccination strategy was adopted from 2003–2004 to 2010–2011 and, thereafter, the PPR-CP vaccination strategy was implemented up to 2025–26. Hence, for the focussed/routine vaccination period (from 2003–2004 to 2009–2010), the vaccine cost per dose was assumed to be twice the vaccine cost for the control programme vaccination period (2010–2011 to 2025–2026) the vaccine cost was INR 1.8/dose and the vaccination logistics and accessories cost was INR18.2/dose [16]. The vaccination logistics and accessories cost included expenditure for accessories (needles, syringes, etc.); payment for hired vaccinators, vaccine storage, and transportation; the cost of sensitisation and technical workshops, extension awareness materials, programme dissemination, and publication; expenditure directed towards the strengthening of district diagnostic laboratories, staff salaries, etc. [16]. The total vaccination cost for the respective year was calculated as follows.

(3) $$C_{v}=\sum_{i=1}^{n}\left({\mathrm{Vp}}_{i},{\times \;\mathrm{Vc}}_{i}\right)$$

where Cv = total cost of vaccination (USD million), VPi = the proportion of sheep and goats vaccinated in the ith year (%); Vci = cost of vaccination (including the vaccine cost and the vaccination logistics and accessories cost per dose) per animal in the ith year (USD million), and n = number of years (i = 1, 2, …, n).

All the benefit and cost streams were estimated in INR and converted to millions of USD (One USD = INR 75).

#### 2.6.7. Financial Assessment of PPR Vaccination

The evaluation of the financial benefits of PPR vaccination was based on the benefit:cost ratio (BCR), net present value (NPV), and the internal rate of returns (IRR) as per standard procedure [16,23] under two vaccination plans, designated as plans I and II. Plan I refers to the actual vaccination coverage from 2003–2004 to 2020–2021 and vaccinating 100% risk population from 2021–2022 to 2023–2024 (for three years), followed by 10% need-based vaccination coverage from 2024–2025 to 2025–2026 (for two years). Plan II refers to the actual vaccination coverage from 2003–2004 to 2020–2021 and vaccinating 100% of the naive population during the year 2021–2022, followed by 30% bi-annual coverage from 2022–2023 to 2023–2024 (the two subsequent years) and 10% need based vaccination coverage from 2024–2025 to 2025–2026 (the next two years), as per the PPR-CP plan.

### 2.7. Perspectives of Veterinary Officers on PPR-CP

A survey was conducted among field veterinarians, who are the custodians of implementing the PPR vaccination programme for the sheep and goats in their jurisdictional veterinary hospitals/dispensaries through the support of the Animal Disease Surveillance Scheme (ADSS), Department of Animal Husbandry and Veterinary Services, Government of Karnataka, using the pre-tested schedules. In addition to implementing PPR-CP, field veterinarians also implement other disease prevention programmes and animal treatment and extension activities. The schedules were mailed to the 176 veterinarians working in veterinary hospitals/dispensaries in the state via e-mail and 62 responded to the survey. The survey schedule comprised statements about the planning and rollout of the PPR control programme in the state, the performance of the functional components of the programme, and the respondent’s opinions regarding its improved implementation. The statements were assessed through a two-point assessment (Yes/No) and a three-point Likert scale (Disagree (SD), Neutral (N), and Agree (A)) with positive and negative statements. The frequencies were calculated based on the responses of the field veterinarians to various statements about PPR control and eradication in Karnataka state.

### 2.8. Statistical Analysis

Descriptive statistics and a two-sample z-test for proportions were used to test the significant differences in terms of diagnosed and dead cases in sheep and goats in the sample farms between survey I (2016–2017) and survey II (2018–2019). These were carried out using SPSS version 22.0 (SPSS Inc., Chicago, IL, USA).

## 3. Results

The results obtained from the secondary data on the reported PPR outbreaks and vaccination coverage in Karnataka state and the results of the cross-sectional primary surveys undertaken in the sampled districts are presented below.

### 3.1. Reported Outbreaks and PPR Vaccination Coverage

**Figure 2:**
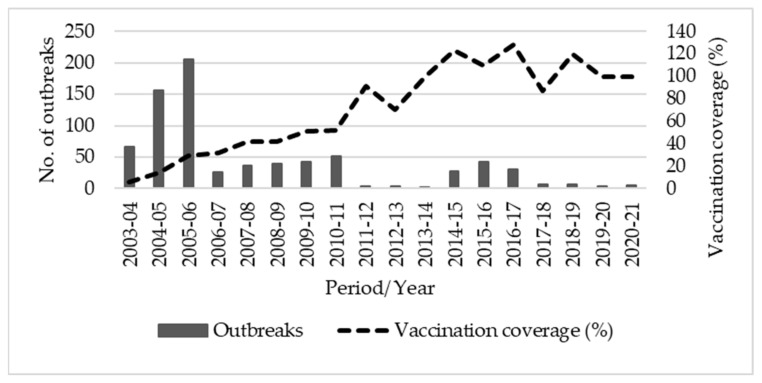
Reported PPR outbreaks and vaccination coverage in Karnataka between 2003–2004 and 2020–2021.

Karnataka practiced focussed vaccination from 2003–2004 to 2010–2011. The vaccination coverage increased from 6% during 2003–2004 to 51% during 2009–2010, whereas after the implementation of PPR-CP in 2010–2011, the vaccination coverage increased consistently and reached 100% in the period from 2014–2015 to 2020–2021, except during 2017–2018 (Figure 2). In consonance with this increased vaccination coverage, the reported PPR outbreaks in the state declined significantly (Figure 2).

### 3.2. Socio-Economic Characteristics of Sheep- and Goat-Rearing Farmers

The socio-economic characteristics of flocks surveyed in survey I and survey II are presented in Table 1. The median ages of farmers rearing sheep and goats during the two surveys were 49 and 46, respectively. The majority of the farmers were illiterate, and the majority of them were small farmers having less than 2.0 ha in land holdings. A considerable proportion of surveyed farmers were landless and they depended entirely on sheep and goats for their livelihoods. The observed average numbers of sheep and goats reared/flock sizes were 40 and 55 during survey I and survey II, respectively (Table 1). The majority (40%) of the sample farmers’ nominal incomes were less than USD 715 during 2016–2017, whereas the majority (52%) of the sample farmers’ nominal incomes were between USD 715 and USD 1429 during 2018–2019. The other socio-economic details of the surveyed flocks in different surveyed districts in Karnataka state during survey I and survey II are presented in Table 1.

### 3.3. PPR Incidence in the Surveyed Flocks

**Figure 3:**
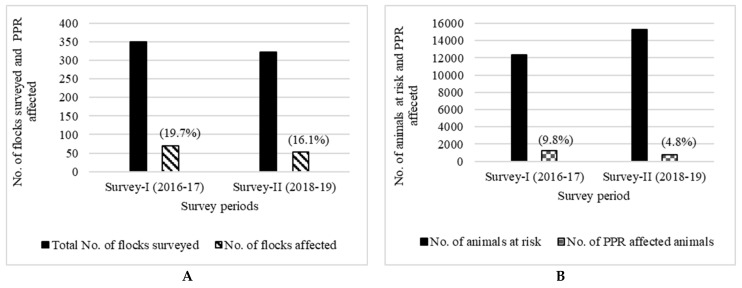
Number of flocks surveyed and proportion of flocks infected with PPR (**A**) and animals at risk and animals infected with PPR (**B**) in survey I and survey II in Karnataka.

The PPR-affected flocks and the disease incidence in sheep and goats among the sample flocks during surveys I and II are presented in Figure 3. The proportion of PPR-affected flocks among the surveyed sheep and goat flocks was 19.7% and 16.1%, respectively (Figure 3A), whereas the PPR incidence at the animal level was 9.8% and 4.8% in survey I and survey II, respectively (Figure 3B).

### 3.4. Distribution of Incidence, Mortality and Case Fatality Rate (CFR)

The age- and sex-wise distributions of PPR incidence, mortality, and CFR in sheep and goats in survey I and survey II are presented in Table 2. The PPR incidence in sheep and goats was 9.8% in survey I, whereas it was 4.8% in survey II. In sheep, age-wise and sex-wise comparisons of incidence showed a significant difference (*p* < 0.01) between the two surveys, whereas, in goats, we observed a significant difference in all the age groups (< 6 months (z = 5.65, *p* < 0.01), 6–12 months (z = 2.52, *p* < 0.1) and > 1 year (z = 5.43, *p* < 0.01)), whereas a non-significant difference was observed in the sex-wise comparison between the two surveys. In sheep, age-wise and sex-wise comparisons of mortality levels showed a significant difference between two surveys, whereas, in goats, a significant difference was observed in the group < 6 months old (z = 6.75, *p* < 0.01) and the 6–12 month old (z = 2.62, *p* < 0.01) group. Furthermore, the CFR was lower in survey I, compared to survey II (Table 2).

### 3.5. Estimated Loss of PPR

The various components of the farm-level loss per animal in surveys I and II are presented in Table 3. The estimated mortality loss ranged between USD 30.1 and USD 128.6 per animal, depending on the severity of the disease in the respective years and the distress sale loss ranged between USD 19.6 and USD 101.8. The body-weight reduction, treatment cost, and opportunity cost of labour ranged between USD 1.7 and USD 7.4, USD 0.3 and USD 7.3, and USD 0.2 and USD 8.3 during the two surveys, respectively. The estimated loss components in sheep and goats independently during the two surveys are presented in Table 3.

### 3.6. Financial Viability of PPR Vaccination

The results of the financial viability assessment of PPR vaccination in Karnataka under the best-, base-, and worst-case PPR incidence scenarios of 15%, 20%, and 25%, respectively, under two methods of vaccination plans are presented in Supplementary Tables S2 and S3. The estimated BCR under the best-, base-, and worst-case incidence scenarios were 18.36:1, 23.45:1, and 28.55:1, whereas the NPV was USD 931.97 million, USD 1205.75 million, and USD 1479.52 million, respectively, under vaccination plan I (Supplementary Table S2). Under vaccination plan II, the estimated BCR was 19.65:1, 25.11:1, and 30.57:1 and the NPV was USD 935.51 million, USD 1209.29 million, and USD 1483.07 million in the best-, base-, and worst-case incidence scenarios, respectively (Supplementary Table S3). Remarkably, an IRR of 412% was observed in all three incidence level scenarios with the two methods of vaccination plans (Supplementary Tables S2 and S3).

### 3.7. Perspective of Field Veterinarians on PPR-CP

#### 3.7.1. Planning and Rollout of PPR-CP

The perceptions of veterinarians with regard to the planning and rollout of PPR-CP in Karnataka state are presented in Table 4. The majority (93.5%) of the surveyed field veterinarians perceived that the PPR-CP had been planned well in the state. For the statement regarding the ‘proper coordination of various programme functionaries’, although the majority agreed on the existence of co-ordination, around 19% remained neutral. Regarding the negative statement concerning the weak support from local Panchayat authorities, the 42% level of agreement indicates that there is still scope for fostering support from the local authorities. Regarding the statement that a ‘delay in the release of funds affected the PPR-CP implementation’, 26% agreed and 42% remained neutral, indicating that there were delays in the release of funds, which in turn affected the implementation of PPR-CP.

#### 3.7.2. Performance of Functional Components of PPR-CP

The majority (82%) of veterinarians perceived that the required quantity of PPR vaccine doses was supplied on time and 72% were able to cover the targeted population in the stipulated time. Regarding the statement on the ‘availability of cold-chain facilities’, 86% of the veterinarians perceived that good facilities were available (Table 5).

#### 3.7.3. Opinions of the Veterinarians on Improving the Implementation of PPR-CP

The majority (84%) of the veterinarians opined on the need for training regarding vaccine efficacy and effectiveness and 77% opined that provision of funds, mainly for mobility and contingency expenditure, were necessary for the success of the PPR-CP implementation in the state. The majority (92%) of the field veterinarians opined that intensive awareness meetings for farmers before the implementation of vaccination would benefit the programme considerably (Table 6).

## 4. Discussion

The socio-economic profile of the sampled farmers revealed that the majority of them were illiterate and small farmers (with landholdings < 2.0 ha) and their annual income was <1500 USD. An outbreak of disease in flocks affects the animal asset pattern, as well as financial and social security, and pushes these farmers to transitional poverty. Hence, to ensure the livelihoods of these farmers, it is important to control and prevent the incidence of PPR in their flocks. Karnataka state reported 150 to 200 outbreaks per year during 2004 to 2006 [2,15]. Due to the adoption of PPR vaccination in the state, the numbers of reported outbreaks have declined significantly since 2006–2007. The maximum vaccination coverage during the focussed vaccination period (2003–2004 to 2009–2010) was only 52%, whereas after the implementation of PPR-CP (2010–2011), a significant increase was observed. In consonance with the increased vaccination coverage, the number of reported PPR outbreaks in the state has declined considerably over the years. A similar pattern of a decline in outbreaks due to mass vaccination was observed in the undivided Andhra Pradesh area, which is a contiguous state of Karnataka.

Though there were fewer reported outbreaks in Karnataka in recent years, the two primary surveys conducted here, covering 673 flocks, revealed incidences of 9.8% (survey I) and 4.8% (survey II), respectively. Similar results showing a higher incidence (17.5%) before mass vaccination (Singh et al., 2014) and a lower incidence (0.8%) after a mass vaccination campaign (MVC) were reported in Chhattisgarh state, India [16]. Age-wise and sex-wise comparisons of incidence and CFR revealed a significant difference between the two surveys. The observed PPR incidences during surveys I and II implied that a significant number of animals became infected with PPR across species, age, and sex groups and a considerable burden was being inflicted upon various stakeholders associated with small-ruminant rearing despite the state being a vaccine-adopted state since 2003–2004. This also implies that vaccination coverage in this particular state alone will not be sufficient to eradicate the disease and the implementation of parallel programmes in the neighboring and contiguous states are also equally important as the regular movement of sheep and goats between these states occurs for trade, transit, and for grazing. Though the number of reported outbreaks significantly decreased in Karnataka state after the implementation of the vaccination programme, in order to obtain a further definitive decline and disease eradication by 2025–2026 through ‘mass vaccination’ in the state, in consonance with the government of India’s PPR eradication plan, an appropriate strategy covering vaccination in all the contiguous regions or epi-systems—mainly in the regions where the maximum level of animal movement is observed between the states—is warranted.

The observed variations in the estimated flock-level losses due to PPR in the surveyed years could be due to differences in the risk population, the number and severity of outbreaks, the age and sex compositions of the diseased flocks, the vaccination coverage in the preceding years, and the time and season of outbreaks. These findings are also in agreement with those of earlier reports [16]. Furthermore, the sensitivity analysis for the various disease incidence scenarios under the two vaccination strategies indicated high gains even under the low-incidence scenario (BCR 18.36 and 19.65; NPV 931.97 and 935.51; and IRR 412% under vaccination plan I and plan II, respectively). A previous study conducted in Chhattisgarh state, India, reported BCRs of 4.9:1, 12.4:1, and 13.5:1 under low, medium, and high PPR incidence levels, respectively, for a 100% yearly vaccination strategy and BCRs of 13.7:1, 34.7:1, and 37.8:1 for a five-year vaccination cycle (100% vaccination in the first year, followed by 30% coverage for three years and need-based coverage in the fifth year) [16]. A study on the economic feasibility of the PPR control programme using the PPR vaccine at the national level, which used an economic surplus model [24], revealed a significant NPV, IRR, and BCR, with values of USD 6988 million (at 1 USD= INR 70), 119%, and 123:1, respectively. Furthermore, a study using a dynamic herd model estimated a BCR of 12.0 for adopting a 5-year period vaccination cycle in Niger [25]. These results imply that PPR vaccination is economically viable and generates more outflows (benefits) than inflows (costs). However, if the Karnataka state intends to eradicate PPR in consonance with the government of India’s plan by 2025–2026, in addition to vaccination, other bio-security measures also need to be promoted in order to reduce the PPR incidence to zero.

The perception of the veterinarians regarding the PPR-CP’s rollout revealed that the majority of the veterinarians concurred with the various activities implemented in Karnataka state, viz., the control programme was planned well, the vaccination programme was accepted by the farmers, proper coordination existed between various functionaries of PPR-CP, and they agreed upon the effectiveness of extension services. Furthermore, regarding the performance of the functional components of the control programme, the majority of the veterinarians perceived that vaccines were available on time in sufficient quantities, they could vaccinate the targeted animals on time, storage and cold-chain facilities were available, and they could manage the outbreaks on time. Though the majority of veterinarians expressed positive opinions on the existing PPR control plan, including its rollout and the performance of the various functional components implemented in the state, a few of the veterinarians disagreed and some remained neutral towards the plan per se, as well as towards the level of coordination between the functionaries, the availability of funding, and the programme’s acceptance by farmers. The veterinarians’ opinions regarding the improvement of the existing control programme included imparting training on vaccine efficacy and effectiveness, increasing the provision of funds for various activities, and increased coordination with various authorities. This implies that there is scope for improvement in the existing programme. Furthermore, since India is planning to eradicate PPR by 2025–2026, all facets of the programme need to be revisited, revised, implemented, and monitored on a regular basis and, more importantly, the perspectives of the important ‘gatekeepers’ in the PPR eradication programme, such as field veterinarians, need to be included in the current programme. Although the availability of effective vaccines, diagnostic tests, veterinary infrastructure, and technical manpower were what prompted the government to initiate PPR control and eradication efforts in small ruminants in India [26], it may be difficult to achieve the eradication target unless shortfalls in the existing programme are addressed. Previous studies also reported the need for strengthening of the veterinary infrastructure, vaccine production, disease diagnoses, and surveillance measures, as well as faster notification in order to achieve PPR disease eradication in Nigeria [27] and in Karnataka [28]. Furthermore, a study on the eradication and control of animal diseases [29] revealed that a combination of measures may be employed to avoid the spread of disease from infected animals to clean animals and their success is dependent on a variety of factors, including the strength and capacity of veterinary services, cross-border efforts for disease vaccination and disease surveillance, political will and support, diagnostic facilities, and financial support. Furthermore, another obstacle that will limit the success of the eradication efforts in India is the unabated movement of animals between and within states for trade and transit, as well as for migration in search of fodder.

Vaccination is an important palliative method to prevent infectious diseases [30]. However, voluntary efforts by farmers to vaccinate their animals on a regular basis are limited in developing countries such as India and hence all the stakeholders in the livestock sector, including farmers, need to be involved for the success of the eradication programme that is planned in India. The results of this study provide evidences and necessary directions for the up-scaling of this strategy in similar socio-economic environments with the aim of eradicating PPR from India and the world by 2030 [16].

At a broader level, the study provides evidence on the operational and financial feasibility of PPR-CP, as implemented in Karnataka state, India. However, the results of the present study need to be visualised with certain limitations, for instance, PPR was confirmed based on the clinical signs observed by farmers and triangulated with those of field veterinarians in the respective jurisdictions and was not based on laboratory confirmation; we also did not consider concomitant diseases; only major farm-level costs were considered in the financial assessments; and only 62 field veterinarians who responded to the survey were considered in the study.

## 5. Conclusions

Though Karnataka state followed a ‘focussed vaccination’ strategy after 2003–2004 and a ‘mass vaccination’ strategy after 2010–2011 under the PPR-CP plan, the disease continues to persist in the state. If the state and the country plan to eradicate PPR, the revisiting of the existing programme is warranted. Furthermore, strong facilitation measures are needed from the federal government for the effective implementation of the eradication programme in these states and to ensure effective coordination between the states. Despite long years of vaccination against PPR, the benefits outweigh the costs, mainly because of the low cost of the vaccine (USD 2.4/100 doses). However, developing countries such as India cannot afford to extend the current control strategies and continue to observe incidences of PPR. Hence, a strong mass vaccination programme that does not exclude a single susceptible animal from vaccination for a considerable period is necessary. Furthermore, the migration routes, trade routes, state borders, and high-risk regions within the states need to be covered during vaccination drives to eradicate the disease. Furthermore, the syndromic surveillance and attending the outbreaks on time, as well as stamping out policy, if needed with compensation to farmers need to be implemented in the final stage of the eradication process.

## Figures and Tables

**Table 1:** General characteristics of sample farms in Karnataka.

Particulars	Unit	Survey I (2016–2017)				Survey II (2018–2019)			
		Districts			Pooled	Districts			Pooled
		Kolar	Bangalore Rural	Bagalkote		Chikkaballapur	Kalaburagi	Bidar	
No. of farms/flocks surveyed	No.	200	90	60	350	205	44	74	323
Age of the farmers (Average)	Years	50	51	44	49	48	45	43	46
Family size (Median)	No.	6	5	8	6	5	6	5	5
**Education**									
Illiterate	No.	101 (51.0)	29 (32.2)	39 (65.0)	205 (58.6)	141 (38.1)	37 (84.1)	60 (81.1)	238 (73.7)
Primary	No.	40 (19.8)	9 (10.0)	7 (11.7)	39 (11.1)	22 (29.3)	4 (9.1)	5 (6.8)	31 (9.6)
High school	No.	40 (19.8)	37 (41.1)	12 (20.0)	80 (22.9)	32 (32.7)	2 (4.6)	5 (6.8)	39 (12.1)
College and above	No.	19 (9.4)	15 (16.7)	2 (3.3)	26 (7.4)	10 (0)	1 (2.3)	4 (5.4)	15 (4.6)
**Land holdings**									
Landless	No.	51 (25.5)	14 (15.5)	18 (30.0)	81 (23.1)	32 (15.6)	27 (61.4)	45 (60.8)	104 (32.2)
Small (< 5 Acre)	No.	140 (70.0)	68 (75.6)	21 (35.0)	229 (65.4)	124 (60.5)	13 (29.6)	24 (32.4)	161 (49.9)
Medium (5 to 10 Acre)	No.	8 (4.0)	6 (6.7)	15 (25.0)	30 (8.6)	41 (20)	3 (6.8)	4 (5.4)	48 (14.9)
Large (> 10 Acre)	No.	1 (0.5)	2 (2.2)	6 (10.0)	10 (2.9)	8 (3.9)	1 (2.3)	1 (1.4)	10 (3.1)
**Income levels (USD)**									
< 715	No.	78 (39.0)	34 (37.8)	10 (16.7)	122 (34.9)	51 (24.9)	0 (0)	17 (22.9)	68 (21.1)
715–1429	No.	73 (36.5)	36 (40.0)	34 (56.7)	141 (40.3)	135 (65.9)	9 (20.5)	26 (35.1)	170 (52.6)
1429–2858	No.	33 (16.5)	15 (16.7)	16 (26.7)	64 (18.3)	18 (8.8)	15 (34.1)	15 (20.3)	48 (14.9)
> 2958	No.	18 (9.0)	5 (5.6)	0 (0.0)	23 (6.6)	1 (0.5)	20 (45.5)	16 (21.6)	37 (11.5)
Total No. of sheep and goats in surveyed farms/flocks	No.	4070	1970	6297	12,337	7765	2649	4897	15,311
Average No. of sheep and goats per farm/flock	No.	27	22	108	40	38	60	66	55
Total No. of sheep and goats sold per year	No.	1706	599	1843	4148	1825	1033	1371	4229
Average No. of sheep and goats sold per year per farm/flock	No.	9	7	31	12	9	23	19	17

Note: Figures in the parenthesis indicate percentages of the total. One USD = INR.70.

**Table 2:** Distribution of morbidity and mortality in sheep and goats due to PPR in Karnataka.

Groups	Category	No. of Surveyed Animals/Animals at Risk		No. of Affected Animals/ Incidence (Morbidity)			No. of Death Cases (Mortality)			CFR (%)	
		2016–2017	2018–2019	2016–2017	2018–2019	*z-*Value |z|	2016–2017	2018–2019	*z-*Value |z|	2016–2017	2018–2019
Animal/species Type	Sheep	10,006	9794	1036 (10.4)	279 (2.8)	21.20 ***	455 (4.5)	207 (2.1)	9.52 ***	43.9	74.2
	Goats	2331	5517	169 (7.3)	460 (8.3)	1.62 ^NS^	81 (3.5)	388 (7)	6.07 ***	47.9	84.3
	Total	12,337	15,311	1205(9.8)	739 (4.8)	15.97 ***	536 (4.3)	595 (3.9)	1.91 *	44.5	80.5
**Sheep**											
Age-wise	<6 months	2112	2812	336 (15.9)	137 (4.9)	13.01 ***	204 (9.7)	113 (4)	7.98 ***	60.7	82.5
	6–12 months	947	3426	108 (11.4)	102 (3)	10.73 ***	30 (3.2)	69 (2)	2.11 *	27.8	67.6
	>1 year	6947	3556	592 (8.5)	40 (1.1)	15.08 ***	221 (3.2)	25 (0.7)	7.94 ***	37.3	62.5
Sex-wise	Male	2029	3939	328 (16.2)	134 (3.4)	17.47 ***	153 (7.5)	99 (2.5)	9.14 ***	46.6	73.9
	Female	7977	5855	708 (8.9)	145 (2.5)	15.45 ***	302 (3.8)	108 (1.8)	6.65 ***	42.7	74.5
**Goats**											
Age-wise	<6 months	633	1476	43 (6.8)	234 (15.9)	5.65 ***	28 (4.4)	217 (14.7)	6.75 ***	65.1	92.7
	6–12 months	267	1830	9 (3.4)	139 (7.6)	2.52 *	5 (1.9)	104 (5.7)	2.62 ***	55.6	74.8
	>1 year	1431	2211	117 (8.2)	87 (3.9)	5.43 ***	48 (3.4)	67 (3)	0.54 ^NS^	41	77
Sex-wise	Male	631	2182	61 (9.7)	227 (10.4)	0.53 ^NS^	30 (4.8)	192 (8.8)	3.32 ***	49.2	84.6
	Female	1700	3335	108 (6.4)	233 (7)	0.45 ^NS^	51 (3)	196 (5.9)	4.47 ***	47.2	84.1
**Sheep + Goats**											
Age-wise	< 6 months	2745	4288	379 (13.8)	371 (8.7)	6.83 ***	232 (8.5)	330 (7.7)	1.14 ^NS^	61.2	88.9
	6–12 months	1214	5256	117 (9.6)	241 (4.6)	6.94 ***	35 (2.9)	173 (3.3)	0.73 ^NS^	29.9	71.8
	> 1 year	8378	5767	709 (8.5)	127 (2.2)	15.51 ***	269 (3.2)	92 (1.6)	5.99 ***	37.9	72.4
Sex-wise	Male	2660	6121	389 (14.6)	361 (5.9)	13.44 ***	183 (6.9)	291 (4.8)	4.05 ***	47	80.6
	Female	9677	9190	816 (8.4)	378 (4.1)	13.26 ***	353 (3.6)	304 (3.3)	1.27 ^NS^	43.3	80.4

Figures in parentheses represent percentages. CFR = case fatality rate, *** indicates significance at 1%; * indicates significance at 10%, ^NS^ = non-significant.

**Table 3:** Components of estimated loss (per animal) on the effects of PPR on sheep and goats in Karnataka.

Parameters	Sheep		Goats		Pooled (Sheep + Goats)	
	2016–2017	2018–2019	2016–2017	2018–2019	2016–2017	2018–2019
Loss due to reduction in body weight (USD)	4.2 (0.4, 1.7–12.5)	4.3 (0.5, 2.9–7.3)	3.9 (0.4, 3.9–7.6)	4.0 (0.4, 2.0–7.4)	4 (0.4, 1.7–12.5)	4.2 (0.4, 2.0–7.4)
Mortality loss (USD)	49.1 (2.4, 25.8–91.9)	56.4 (2.6, 34.3–128.6)	51.2 (2.5, 34.7–99.6)	56.5 (2.5, 42.9–120.0)	50.2 (2.5, 30.1–99.6)	56.5 (2.6, 34.3–128.6)
Distress sale loss (USD)	43.3 (1.9, 26.1–76.5)	0 (0, 0–0)	38.3 (1.5, 15.3–68.9)	84.9 (3.7, 35.7–101.8)	40.8 (1.7, 19.6–76.5)	84.9 (13.7, 35.7–101.8)
Treatment cost (USD)	1.6 (0, 0.3–9.2)	2.6 (0.07, 0.9–7.3)	1.6 (0, 0.3–9.2)	2.6 (0.07, 0.9–7.3)	1.6 (0, 0.3–9.2)	2.6 (0.07, 0.9–7.3)
Opportunity cost of labour (USD)	1.8 (0.1, 0.2–4)	2.4 (0.05, 0.4–8.3)	1.8 (0.1, 0.2–4)	2.4 (0.05, 0.4–8.3)	1.8 (0.1, 0.2–4)	2.4 (0.05, 0.4–8.3)

Figures in parentheses represent standard error and range values.

**Table 4:** Survey results on the perceptions of veterinarians regarding the planning and rollout of the PPR control programme (PPR-CP) in Karnataka (*N* = 62).

Statements	Disagree	Neutral/Undecided	Agree
The PPR vaccination programme has been well planned in your jurisdiction.	4 (6.5, 0.3–12.6)	0 (0, 0–0)	58 (93.5, 87.4–99.7)
Proper coordination exists between various functionaries.	1 (1.6, −1.5–4.7)	12 (19.4, 9.5–29.2)	49 (79, 68.9–89.2)
Funding delays hindered the implementation of PPR-CP.	20 (32.3, 20.6–43.9)	26 (41.9, 29.7–54.2)	16 (25.8, 14.9–36.7)
Weak support from local authority such as the Panchayat was experienced.	20 (32.3, 20.6–43.9)	16 (25.8, 14.9–36.7)	26 (41.9, 29.7–54.2)
The vaccination programme was acceptable for farmers	1 (1.6, −1.5–4.7)	10 (16.1, 7–25.3)	51 (82.3, 72.7–91.8)
Creating awareness about PPR and vaccination for farmers and other functionaries through mass media is effective in its implementation.	1 (1.6, −1.5–4.7)	4 (6.5, 0.3–12.6)	57 (91.9, 85.2–98.7)

Figures in parentheses indicate percentages and 95% confidence intervals.

**Table 5:** Perceptions of veterinarians regarding the performance of the functional components of PPR-CP in Karnataka (*n* = 62).

Statements	Disagree	Neutral/Undecided	Agree
Timely availability of vaccines	4 (6.5, 0.3–12.6)	7 (11.3, 3.4–19.2)	51 (82.3, 72.7–91.8)
Supply of sufficient quantity of vaccines and quality of vaccination materials (syringes, gloves, etc.)	6 (9.7, 2.3–17)	5 (8.1, 1.3–14.8)	51 (82.3, 72.7–91.8)
Able to cover the targeted population within the stipulated time	9 (14.5, 5.7–23.3)	8 (12.9, 4.6–21.2)	45 (72.6, 61.5–83.7)
Appropriate training was imparted to the vaccination team to vaccinate animals at the farmer’s doorstep	10 (16.1, 7–25.3)	9 (14.5, 5.7–23.3)	43 (69.4, 57.9–80.8)
Storage and cold-chain facilities were good	6 (9.7, 2.3–17)	3 (4.8, −0.5–10.2)	53 (85.5, 76.7–94.3)
Veterinarians were able to manage the reported outbreaks in time	1 (1.6, −1.5–4.7)	8 (12.9, 4.6–21.2)	53 (85.5, 76.7–94.3)

Figures in parentheses indicate percentages and 95% confidence intervals.

**Table 6:** Opinions of veterinarians regarding statements for the improved implementation of PPR-CP in Karnataka (*n* = 62).

Statements	Yes	No
Training should be supplied to veterinarians and para-veterinary workers on the vaccine’s efficacy and effectiveness	52(83.9, 74.7–93.0)	10(16.1, 6.9–25.3)
Provision of funds for mobility, contingencies, etc., is key for success	48(77.4, 67.0–87.8)	14(22.6, 12.2–32.9)
Involving various local authorities in vaccination will strengthen the programme	45(72.6, 61.5–83.7)	17(27.4, 16.3–38.5)
Organising farmer-awareness meetings is one way to make the programme better	57(91.9, 85.2–98.7)	5(8.1, 1.3–14.8)

Figures in parentheses indicate percentages and 95% confidence intervals.

## Data Availability

The data presented in this study are available in the manuscript and in the Supplementary Files.

## Supplementary Materials

The following supporting information can be downloaded at: https://www.mdpi.com/article/10.3390/ani13050778/s1, Table S1: Different interpolated PPR incidence levels under scenarios with vaccination and without vaccination—(low (15%), medium (20%), and high (25%)) from 2003–2004 to 2025–26 in Karnataka. Table S2: Financial viability of the PPR vaccination in Karnataka under vaccination plan I (actual vaccination coverage (from 2003–2004 to 2020–2021) with the vaccination of 100% of the at-risk population for three years (from 2021–2022 to 2023–2024), followed by 10% need-based vaccination coverage for the next two years (from 2024–2025 to 2025–2026)). Table S3: Financial viability of the PPR vaccination in Karnataka under vaccination plan II (actual vaccination coverage (from 2003–2004 to 2020–2021) with the vaccination of 100% of the naive population during the year 2021–2022, followed by 30% bi-annual coverage during the two subsequent years (from 2022–2023 to 2023–2024) and 10% need-based vaccination coverage for the next two years (from 2024–2025 to 2025–2026) as per the PPR-CP plan).
