# Supplementary material for: Towards Eradication of PPR: Disease Status, Economic Cost and Perception of Veterinarians in Karnataka, India
Source: Animals (Basel). 2023 Feb 21;13(5):778. doi: 10.3390/ani13050778 (PMC10000058; doi:10.3390/ani13050778)
Supplement: Supplementary file 1 [file animals-13-00778-s001.zip › animals-2162877-supplementary.pdf]

animals

MDPI

Towards Eradication of PPR: Disease Status, Economic Cost and Perception of Veterinarians in Karnataka, India

Gurrappa Naidu Govindaraj [1*], Vinayagamurthy Balamurugan [1], GB Manjunatha Reddy [1], Revanaiah Yogisharadhya [1], Timmareddy Sreenivasa Reddy [2], Gajalavarahalli Subbanna Naveenkumar [1], Kirubakaran Vinod Kumar [1], Hosahalli Rajanna Chaithra [1], Afrin Zainab Bi [1], Satya Parida [3], Felix Njeumi [3], Parimal Roy [1] and Bibek Ranjan Shome [1]

Supplementary Materials

Table S1. Represents different interpolated PPR incidence levels under *vaccination* and *without vaccination scenario* (low (15%), medium (20%) and high (25%)) from 2003-04 to 2025-26 in Karnataka.

Years	Incidence under with vaccination scenario (%) (A)	Incidence under without vaccination scenario (%) (B)			Difference between incidence under with and without vaccination (C = B − A)		
		Low (15%)	Medium (20%)	High (25%)	Low (15%)	Medium (20%)	High (25%)
2003-04	8	8	8	8	0	0	0
2004-05	8.6	9	9	9	0.4	0.4	0.4
2005-06	9.1	10	10	10	0.9	0.9	0.9
2006-07	9.7	11	11	11	1.3	1.3	1.3
2007-08	10.3	12	12	12	1.7	1.7	1.7
2008-09	10.9	13	13	13	2.1	2.1	2.1
2009-10	11.4	14	14	14	2.6	2.6	2.6
2010-11	12	15	15	15	3.0	3.0	3.0
2011-12	11.6	15	15.3	15.7	3.4	3.7	4.0
2012-13	11.3	15	15.7	16.3	3.7	4.4	5.1
2013-14	10.9	15	16	17	4.1	5.1	6.1
2014-15	10.5	15	16.3	17.7	4.5	5.8	7.1
2015-16	10.2	15	16.7	18.3	4.8	6.5	8.2
2016-17	9.8	15	17	19	5.2	7.2	9.2
2017-18	7.3	15	17.3	19.7	7.7	10.0	12.4
2018-19	4.8	15	17.7	20.3	10.2	12.9	15.5
2019-20	3.8	15	18	21	11.2	14.2	17.2
2020-21	2.9	15	18.3	21.7	12.1	15.5	18.8
2021-22	1.9	15	18.7	22.3	13.1	16.7	20.4
2022-23	1	15	19	23	14.0	18.0	22.0
2023-24	0	15	19.3	23.7	15.0	19.3	23.7
2024-25	0	15	19.7	24.3	15.0	19.7	24.3
2025-26	0	15	20	25	15.0	20.0	25.0

Table S2. Financial viability of the PPR vaccination in Karnataka under vaccination plan –I (actual vaccination coverage (from 2003-04 to 2020-21) with vaccinating 100% risk population for three years (from 2021-22 to 2023-24) followed by 10% need based vaccination coverage for next two years (from 2024-25 to 2025-26)).

Year	PPR incidence under without vaccination (%)			PPR incidence under with vaccination (%) (D)	Difference in incidence (%)			Vaccine coverage (%)	Estimated avoided average loss undue different scenario corrected for vaccine effectiveness (USD in million)			Total vaccination cost (USD in million)	Financial viability measures		
	Low (A)	Medium (B)	High (C)		Low (A − D)	Medium (B − D)	High (C − D)		Low	Medium	High		Low	Medium	High
2003-04	8	8	8	8.00	0.00	0.00	0.00	6	0.00	0.00	0.00	0.25			
2004-05	9	9	9	8.57	0.43	0.43	0.43	14	1.25	1.25	1.25	0.47			
2005-06	10	10	10	9.14	0.86	0.86	0.86	29	2.78	2.78	2.78	1.13			
2006-07	11	11	11	9.71	1.29	1.29	1.29	32	4.62	4.62	4.62	1.35			
2007-08	12	12	12	10.29	1.71	1.71	1.71	42	6.84	6.84	6.84	1.73			
2008-09	13	13	13	10.86	2.14	2.14	2.14	42	8.65	8.65	8.65	1.74			
2009-10	14	14	14	11.43	2.57	2.57	2.57	51	10.52	10.52	10.52	2.13			
2010-11	15	15	15	12.00	3.00	3.00	3.00	52	12.43	12.43	12.43	2.16			
2011-12	15	15.33	15.67	11.63	3.37	3.70	4.03	91	14.12	15.52	16.92	3.51			
2012-13	15	15.67	16.33	11.27	3.73	4.40	5.07	70	15.86	18.70	21.53	0.74			
2013-14	15	16	17	10.90	4.10	5.10	6.10	100	18.43	22.92	27.42	4.88			
2014-15	15	16.33	17.67	10.53	4.47	5.80	7.13	123	21.24	27.58	33.92	1.41			
2015-16	15	16.67	18.33	10.17	4.83	6.50	8.17	110	24.31	32.69	41.07	2.54			
2016-17	15	17	19	9.80	5.20	7.20	9.20	128	27.67	38.31	48.95	2.96			
2017-18	15	17.33	19.67	7.30	7.70	10.03	12.37	87	43.34	56.47	69.60	3.31			
2018-19	15	17.67	20.33	4.80	10.20	12.87	15.53	120	60.72	76.60	92.48	4.58			
2019-20	15	18	21	3.84	11.16	14.16	17.16	100	70.22	89.09	107.97	2.31			
2020-21	15	18.33	21.67	2.88	12.12	15.45	18.79	100	80.34	102.43	124.53	2.44			
2021-22	15	18.67	22.33	1.92	13.08	16.75	20.41	100	91.34	116.94	142.54	4.28			
2022-23	15	19	23	0.96	14.04	18.04	22.04	100	103.28	132.71	162.13	4.38			
2023-24	15	19.33	23.67	0.00	15.00	19.33	23.67	100	116.24	149.83	183.41	4.48			
2024-25	15	19.67	24.33	0.00	15.00	19.67	24.33	10	122.46	160.56	198.66	0.46			
2025-26	15	20	25	0.00	15.00	20.00	25.00	10	129.01	172.01	215.01	0.47			
											BCR		18.36	23.45	28.55
											NPV (in USD million)		931.97	1205.75	1479.5
											IRR (%)		412	412	412

Table S3. Financial viability of the PPR vaccination in Karnataka under vaccination plan –II (actual vaccination coverage (from 2003-04 to 2020-21) with vaccinating 100% naive population during the year 2021-22 followed by 30% bi-annual coverage during two subsequent years (from 2022-23 to 2023-24) and 10% need based vaccination coverage for next two years (from 2024-25 to 2025-26) as per the PPR-CP plan).

Year	PPR incidence under without vaccination (%)			PPR incidence under with vaccination (%) (D)	Difference in incidence (%)			Vaccine coverage (%)	Estimated avoided average loss undue different scenario corrected for vaccine effectiveness (USD in million)			Total vaccination cost (USD in million)	Financial viability measures		
	Low (A)	Medium (B)	High (C)		Low (A − D)	Medium (B − D)	High (C − D)		Low	Medium	High		Low	Medium	High
2003-04	8	8	8	8.00	0.00	0.00	0.00	6	0.00	0.00	0.00	0.25			
2004-05	9	9	9	8.57	0.43	0.43	0.43	14	1.25	1.25	1.25	0.47			
2005-06	10	10	10	9.14	0.86	0.86	0.86	29	2.78	2.78	2.78	1.13			
2006-07	11	11	11	9.71	1.29	1.29	1.29	32	4.62	4.62	4.62	1.35			
2007-08	12	12	12	10.29	1.71	1.71	1.71	42	6.84	6.84	6.84	1.73			
2008-09	13	13	13	10.86	2.14	2.14	2.14	42	8.65	8.65	8.65	1.74			
2009-10	14	14	14	11.43	2.57	2.57	2.57	51	10.52	10.52	10.52	2.13			
2010-11	15	15	15	12.00	3.00	3.00	3.00	52	12.43	12.43	12.43	2.16			
2011-12	15	15.33	15.67	11.63	3.37	3.70	4.03	91	14.12	15.52	16.92	3.51			
2012-13	15	15.67	16.33	11.27	3.73	4.40	5.07	70	15.86	18.70	21.53	0.74			
2013-14	15	16	17	10.90	4.10	5.10	6.10	100	18.43	22.92	27.42	4.88			
2014-15	15	16.33	17.67	10.53	4.47	5.80	7.13	123	21.24	27.58	33.92	1.41			
2015-16	15	16.67	18.33	10.17	4.83	6.50	8.17	110	24.31	32.69	41.07	2.54			
2016-17	15	17	19	9.80	5.20	7.20	9.20	128	27.67	38.31	48.95	2.96			
2017-18	15	17.33	19.67	7.30	7.70	10.03	12.37	87	43.34	56.47	69.60	3.31			
2018-19	15	17.67	20.33	4.80	10.20	12.87	15.53	120	60.72	76.60	92.48	4.58			
2019-20	15	18	21	3.84	11.16	14.16	17.16	100	70.22	89.09	107.97	2.31			
2020-21	15	18.33	21.67	2.88	12.12	15.45	18.79	100	80.34	102.43	124.53	2.44			
2021-22	15	18.67	22.33	1.92	13.08	16.75	20.41	100	91.34	116.94	142.54	4.28			
2022-23	15	19	23	0.96	14.04	18.04	22.04	60	103.28	132.71	162.13	2.63			
2023-24	15	19.33	23.67	0.00	15.00	19.33	23.67	60	116.24	149.83	183.41	2.69			
2024-25	15	19.67	24.33	0.00	15.00	19.67	24.33	10	122.46	160.56	198.66	0.46			
2025-26	15	20	25	0.00	15.00	20.00	25.00	10	129.01	172.01	215.01	0.47			
										BCR			19.65	25.11	30.57
										NPV (in USD million)			935.51	1209.29	1483.1
										IRR (%)			412	412	412
